# Pathophysiology of Atrial Fibrillation and Approach to Therapy in Subjects Less than 60 Years Old

**DOI:** 10.3390/ijms25020758

**Published:** 2024-01-07

**Authors:** Antonio Curcio, Rosa Scalise, Ciro Indolfi

**Affiliations:** Division of Cardiology, Department of Medical and Surgical Sciences, Magna Graecia University, 88100 Catanzaro, Italy; rosy.scalise@studenti.unicz.it (R.S.); indolfi@unicz.it (C.I.)

**Keywords:** atrial fibrillation, cardiac arrhythmias, cardiovascular risk, ion channels, intracellular pathways

## Abstract

Atrial fibrillation (AF) is an arrhythmia that affects the left atrium, cardiac function, and the patients’ survival rate. Due to empowered diagnostics, it has become increasingly recognized among young individuals as well, in whom it is influenced by a complex interplay of autoimmune, inflammatory, and electrophysiological mechanisms. Deepening our understanding of these mechanisms could contribute to improving AF management and treatment. Inflammation is a complexly regulated process, with interactions among various immune cell types, signaling molecules, and complement components. Addressing circulating antibodies and designing specific autoantibodies are promising therapeutic options. In cardiomyopathies or channelopathies, the first manifestation could be paroxysmal AF; persistent forms tend not to respond to antiarrhythmic drugs in these conditions. Further research, both in vitro and in vivo, on the use of genomic biotechnology could lead to new therapeutic approaches. Additional triggers that can be encountered in AF patients below 60 years of age are systemic hypertension, overweight, diabetes, and alcohol abuse. The aims of this review are to briefly report evidence from basic science and results of clinical studies that might explain the juvenile burden of the most encountered sustained supraventricular tachyarrhythmias in the general population.

## 1. Introduction

Atrial fibrillation (AF) is the sustained supraventricular tachy-arrhythmia mostly encountered in the clinical practice. Since it has been associated with a series of negative outcomes, including death, acute coronary syndromes, heart failure (HF), hospitalization, and stroke, recent guidelines recommend taking the pulse occasionally in all patients over 65 years [1] to start follow-up programs or therapies if needed. While such an approach represents an effective clinical strategy that considers the belief that AF is the new pandemic of industrialized countries, its negative impact on an ageing population and relative costs for health systems must be reduced [2]. On the other hand, patients below 60 years of age might not be considered at risk. Such an assumption is untrue for several reasons, spanning from the fact that the main risk score for thromboembolism, the CHA_2_DS_2_VASc score that includes congestive heart failure, hypertension, age ≥ 75 (doubled), diabetes, stroke (doubled), vascular disease, and sex category, considers at maximum one point for patients aged over 65 years without taking into account the remaining risk factors, and other studies clearly show that the overt HF recognizes roots in an early stage of tachy-cardiomyopathy which might have been established years before, perhaps below the age of 60 years [3]. Moreover, current diagnostic strategies based on wearable devices have augmented the number of patients diagnosed with asymptomatic AF (the so called “subclinical AF”) and such diagnostics are more widespread in younger patients, since among people below 60 years age, the highest rates of digital literacy are observed. Therefore, the aim of this review will be to describe the main determinants for AF to occur in younger patients, the pathophysiologic backgrounds predisposing AF in patients below 60 years, and current approaches to therapy.

## 2. Autoimmune Mechanisms of AF

**Figure 1:**
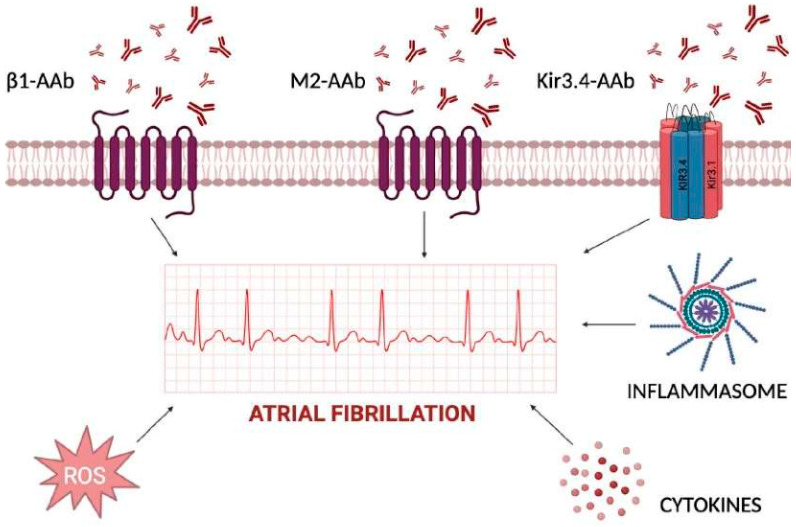
Inflammatory determinants of atrial fibrillation acting at extra- and intra-cellular levels. The cardiomyocyte plasma membrane is regulated by several channels, with either transmembrane-spanning domains or pore-forming domains, and further signal transduction into cytoplasm for activation of cytokines, inflammasome, and reactive oxygen species (ROS). Extracellular autoantibodies can trigger each one of the reported mechanisms by changing atrial cardiomyocytes action potential duration and inducing atrial fibrillation.

The shift from the main role of triggers based upon anatomical and genetic factors toward autoantibodies that interfere with ion channels and receptors currently represents an area for intense investigation. Autoantibodies induce cell death by targeting various cardiac cellular receptors that modulate the autonomic nervous system and related signaling pathways. In particular, autoantibodies against β_1_-adrenergic receptors or M2-acetylcholine muscarinic receptors have been considered potential contributors to the pathogenesis of AF, acting as sympathomimetic and parasympathomimetic agonists, respectively [4]. Animal studies have shown that these autoantibodies can contribute to atrial structural and electrophysiological remodeling, increasing atrial arrhythmogenicity and creating a favorable substrate for AF development (Figure 1). It has been shown that the β_1_-adrenergic receptor autoantibodies (β_1_-AAb) contribute to the development of autoimmunity-associated AF by targeting the functional domain on the second extracellular loop of the receptor itself. In vivo models revealed that β_1_-AAb contribute to atrial electrical instability through the activation of Ca^2+^/calmodulin-dependent protein kinase II (CaMKII) and phosphorylation of the ryanodine receptor (RyR2) [5]. These mechanisms prolong transient calcium refractoriness and promote arrhythmogenic and spatially discordant atrial alternans. 

The involvement of circulating autoantibodies against M2-muscarinic acetylcholine receptors (anti-M2-R) in AF has been studied in preclinical models [6] and in humans as well [7]. The increased expression of tumor growth factor (TGF)-β1 and connective tissue GF (CTGF) in left atrial appendage tissues correlates with the severity of atrial fibrosis [8].

Additional proof about the role of autoantibodies in AF has come from peptide microarrays which demonstrated the targeting of the extracellular site of the Kir3.4 protein in patients affected by AF [9].

Kir3.4 and its specific activated potassium current, IKACh, mediate the negative chronotropic effect of the parasympathetic nervous system [10]; during AF, there is an increased likelihood of opening constitutively active IKACh channels, which escape muscarinic cholinergic regulation. These phenomena, along with altered calcium handling, atrial contractility, and conduction, contribute to the formation of reentrant circuits which maintain AF [11]. To this regard, the maintenance of AF is favored by the cyclic nature of inflammatory/oxidative stress, which begets AF [12,13].

Glutathione and cysteine are biomarkers of increased risk of AF [14], since both remove radical oxygen species (ROS) which can directly influence cardiac ion channels. ROS act by: (i) triggering late Na^+^ current [15]; (ii) impairing calcium handling through oxidation of CAMKII [13]; and (iii) affecting the phosphorylation of RyR2 which causes early and delayed afterdepolarizations and reentry circuit formation [16].

Systemic inflammatory biomarkers including high sensitivity C-reactive protein (hs-CRP), tumor necrosis factor α (TNF-α), and interleukin (IL)-6, have been identified as risk factors for both the onset and recurrence of AF after transcatheter ablation [13]. Their mechanisms of action in AF have been linked to NLR family pyrin domain-containing 3 (NLRP3) inflammasome, which is abundant in atrial samples from patients with paroxysmal and persistent AF [17,18,19]. The inflammasome appears to promote ectopic activation contributing to the formation of AF-related substrates, including RYR2 and ultrarapid K channels [13]. 

## 3. Early CV Diseases and Atrial Fibrillation in the Young

Comorbidities associated with AF, such as excess weight, physical inactivity, sleep respiratory disorders, diabetes mellitus, and systemic hypertension, potentially lead to further perpetuation of the AF substrate [20].

Regarding obesity, several mechanistic studies suggest that the stearic acid present in pericardial and epicardial fat can impair ionic channels [21]. In addition, obesity increases the risk of hypertension and diastolic dysfunction, with augmented AF susceptibility via stretch-activated left atrial channels [22,23]. The risk of developing obesity and cardiovascular disease can be reduced with physical activity. 

Hypertension is also seen in association with obesity and is related to alterations in hemodynamics and increased ventricular afterload, which lead to cardiac hypertrophy and left atrium overload [1,2,15]. 

Obstructive sleep apnea (OSA) is characterized by recurrent short apneic episodes due to pharyngeal airway collapse. OSA promotes the occurrence and recurrence of AF, which is the most common clinically observed form of arrhythmia, due to the repetitive cycles of intermittent hypoxia causing an imbalance of cardiac autonomic modulation. Over time, OSA can also promote prolonged systemic inflammation, a prothrombotic state, atrial fibrosis, and electrical remodeling [24,25].

Although data on precise mechanistic pathways for diabetes mellitus (DM) remain limited, studies suggest that electrical and cardiac remodeling along with hyperglycemia-induced fibrotic change likely contribute to increased AF susceptibility. N-glycosylation involves the co- and post-translational addition of complex oligosaccharide structures (glycans) to proteins, influencing their structure, function, and processing, and are crucial for many biological processes, including cell signaling and recognition [26]. Recently, it has been shown that semaglutide, a glucagon-like peptide-1 receptor agonist that reduces the risk of adverse cardiovascular events in patients with diabetes, can lessen the cardiovascular risk associated with overweight and obese patients in the absence of diabetes as well [27]. 

Tobacco addiction is a major risk factor for AF; either direct cigarette smoking or heat-not-burn tobacco products may increase vulnerability to AF through direct effect of nicotine which favors sympathetic neurotransmission, as well as indirectly by ensuing atrial fibrosis [28].

## 4. Inherited Arrhythmogenic Diseases and AF

Inherited arrhythmogenic diseases (IADs) include cardiomyopathies, resulting from mutations in genes encoding specific structural proteins, and channelopathies characterized by alterations in transmembrane channel regulating action potential. Both disorders are associated with atrial remodeling, histological changes, and alterations in atrial action potential features that may increase the risk of AF. Each of these inherited diseases present unique pathogenetic features.

### 4.1. Long QT Syndrome

Long QT Syndrome (LQTS) is a primary electrical disorder characterized by the prolongation of the repolarization process, as evidenced by the prolongation of the QT interval on the electrocardiogram (ECG). This condition is linked to potentially life-threatening ventricular arrhythmias and, although more commonly observed in childhood, it carries a long-term risk of such arrhythmias persisting into adulthood [29,30,31,32,33].

Given that most ion channels are present in both atrial and ventricular myocardium, LQTS may also impact atrial electrophysiology and be associated with an increased risk of AF [Table 1] [34,35,36,37].

**Figure 2:**
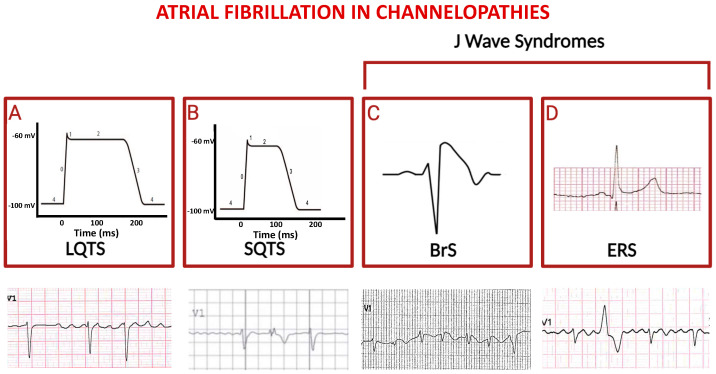
Schematic representation of principal channelopathies in which atrial fibrillation occurs. The schemes in (**A**) and (**B**) indicate prolonged versus shortened cardiomyocyte action potential duration (phases are numbered from 0 to 4) in LQTS and in SQTS, respectively; third and fourth squares show paradigmatic ST-segment upward shifts in right precordial lead (Brugada syndrome, (**C**)) and in peripheral lateral I lead (Early Repolarization Syndrome, (**D**)). Electrocardiographic tracings reported below depict atrial fibrillation in the context of the above indicated diseases (ventricular extra-systolic beats appear in second and fourth ECGs).

In a large group of LQTS patients, a significant association between the LQT3 genotype and an increased risk of early AF has been observed [38,39,40,41,42,43]. Conversely, patients with LQT2 have a much-reduced risk of developing AF throughout their lives, suggesting a potential protective effect of LQT2 mutations in this context [44,45,46]. According to the proposed hypothesis, LQT2 mutations may prolong the repolarization phase in atrial cardiomyocytes, similar to the effects of some class III antiarrhythmics used to prevent AF. In contrast, LQT1 mutations may not result in a significant prolongation of the refractory period in atrial myocardium, while prolonging the QT segment (Figure 2A) [47,48,49,50,51].

### 4.2. Short QT Syndrome

The Short QT syndrome (SQTS) is a rare inherited cardiac channelopathy, primarily caused by gain-of function mutations in the genes encoding for potassium channels. This results in abnormally short QT interval and in increased risk of atrial and ventricular arrhythmias [52]. So far, six subtypes of SQTS have been identified, each linked to nine mutations in six distinct genes encoding different cardiac ion channels, with the potassium channels being most frequently affected in all subtypes of the syndrome [Table 1]. The increase in transmural repolarization dispersion and shortening of the repolarization period explain the main features of this syndrome: short atrioventricular effective refractory periods and short QT intervals, which increase susceptibility to ventricular fibrillation, and AF as well (Figure 2B) [53]. Pharmacological therapy can be indicated as an alternative to implantable cardioverter/defibrillator (ICD) in young patients, or when ICD is refused or contraindicated, and to prevent symptomatic AF. In patients with SQTS and recurrent ICD shocks, quinidine has been shown to prevent further ICD discharges, since it reduces AF burden.

### 4.3. J Wave Syndromes

The term “J Wave Syndromes” derives from unique ST-segment elevation on the ECG encountered in Brugada Syndrome (BrS) and in Early Repolarization Syndrome (ERS), both associated with sudden cardiac death (SCD). BrS is an inherited arrhythmia syndrome that accounts for ~20% of SCD cases in young, healthy adults with structurally normal hearts [54,55].

It is electrophysiologically characterized by a typical type 1 ECG pattern displaying a coved ST-segment elevation of at least 2 mm followed by a negative T wave in at least one right precordial lead and by a high incidence of life-threatening ventricular arrhythmias [56,57,58]. The incidence of AF in BrS patients has been reported to range from 11% to 39%, and it is considered an indicator of unfavorable prognosis. Conversely, latent BrS has been observed in young patients with AF without pre-existing conditions and/or known risk factors [59]. The SCN5A gene, representing approximately 20–30% of BrS cases, stands as the sole gene considered clinically validated, due to loss-of-function mutations [60].

Interestingly, the prominence of I*_to_* currents [54,55] within the atria [56,57,58] is thought to contribute to atrial disease [59,60,61,62] and atrial arrhythmias [63,64] in patients with BrS [Table 2].

The investigation of genetic isolates has demonstrated an increase in AF among the phenotypes of the affected members harboring sodium channel gene mutations (Figure 2C) [55]; however, SCD in those cases was associated with malignant ventricular tachy-arrhythmias, which were not predictable according to AF occurrence. Repolarization abnormalities observed in ERS (Figure 2D) may be upsloping, horizontal, or descending. Horizontal and descending ST-segments are associated with a worse prognosis. Early repolarization may indicate increased susceptibility to AF [48,65], although supraventricular tachycardias in ERS patients have been documented with lower prevalence compared to BrS; to this regard, AF catheter ablation successfully reduces the burden of life-threatening ventricular arrhythmias in ERS.

### 4.4. Biatrial Myopathy and Atrial Fibrillation

The heritability of AF has been investigated in depth since the first report of familial atrial fibrillation (FAF) in 1936 [66].

In a Framingham offspring study, it was demonstrated that those who had a parent with a history of AF had a significantly higher likelihood of developing this arrhythmia, with a threefold higher risk in individuals under the age of 75 years. The results of a study involving 5000 Icelanders further highlighted how first-degree relatives of patients with AF had a 1.77 times higher probability of developing this condition compared to the general population [67]. Additionally, an analysis conducted on monozygotic twins estimated that the heritability of AF could reach up to 62% [68]. Of note, FAF is not related solely to ion channel genes [13,15,33,35] and structural protein-encoding genes [39,40,41,43,44], but also to transcriptional regulators genes [46,47,48,67,68,69,70,71].

**Figure 3:**
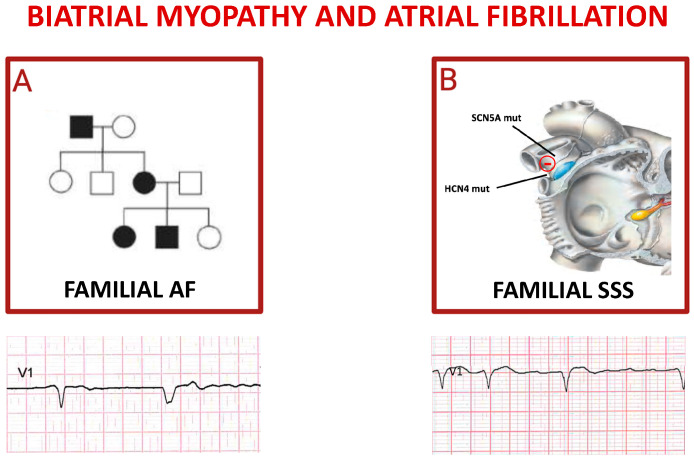
Inherited atrial fibrillations. Panel (**A**) shows autosomal dominant pattern of inheritance of familial atrial fibrillation; the pedigree depicts an affected grandfather (black square) transmitting mutation to one daughter (black circle) who gave birth to a not affected daughter (white circle) and two affected children. The ECG reported below shows a spontaneous QRS complex followed by a paced QRS complex in a patient affected by FAF. Cartoon in (**B**) depicts the main genes whose mutations trigger sick sinus syndrome (SSS), mostly characterized by alternance of brady- and tachy-cardia rhythms in the same subject (see related ECG).

An elegant study started by sequencing GJA5 from resected cardiac tissues and peripheral lymphocytes of 15 patients with idiopathic AF [72]. GJA5 encodes connexin (Cx) 40, a gap junction protein with gene expression restricted primarily to atrial tissue in humans. Cx40 gap junctions play a critical role in mediating atrial conduction through electrical coupling between cells. GJA5 knockout mice have been shown to have increased vulnerability to atrial reentrant arrhythmias [73]. Furthermore, they suggest that sequence variations in potential regulatory regions of genes encoding for Cx40 and Cx43 may increase the risk of AF (Figure 3A) [74].

Recently, many studies investigated the role of cytoskeletal proteins in the pathogenesis of FAF [Table 3] [75,76,77,78,79].

An example is MYL4 which encodes the essential subunit of the myosin light chain, known as atrial light chain1. In vitro experiments in MYL4 mutant zebrafish revealed disruption of sarcomeric structure, atrial enlargement, and electrical abnormalities associated with human AF [80].

The ORBIT-AF registry has highlighted a greater manifestation of symptoms in FAF patients compared to non-affected individuals; however, no significant differences have emerged in terms of AF recurrences, hospitalization rates, complications, and overall mortality [81]. Future studies should examine the effect of gene-environment interactions on a large scale and include participants of diverse ethnic backgrounds, along with a better understanding of the underlying mechanisms of FAF genetic variants. Recently, functional alterations in the sinus node (the so-called sick sinus syndrome, SSS) have been observed in a familial form as a genetic disorder. The most prevalent genes responsible for familial SSS are those encoding SCN5A [29,33,55,61] and hyperpolarization-activated cyclic nucleotide-gated channel (HCN4) (Figure 3B) [49,82].

### 4.5. Hypertrophic Cardiomyopathy 

Hypertrophic cardiomyopathy (HCM) is a relatively common condition that can lead to SCD, even in young individuals, including well-trained athletes. This pathology affects both men and women, irrespective of their ethnicity [83].

HoCM is part of the broader context of hypertrophic cardiomyopathy (HCM), a genetic disease with an autosomal dominant inheritance pattern caused by mutations in sarcomere proteins. A subset of patients with HCM have an obstructed left ventricular outflow tract, which is the hallmark of HoCM [84]. 

The disease is characterized by significant hypertrophy and fibrosis of the subaortic region of the interventricular septum, which is macroscopically evident with a thick septal wall measuring between 13–15 mm and, in some cases, reaching 50 mm. 

**Figure 4:**
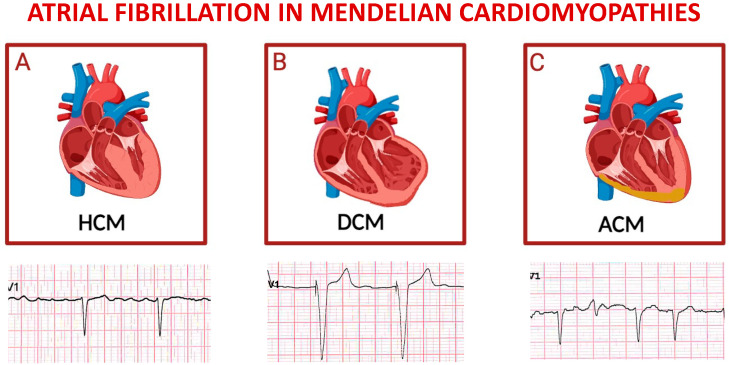
Schematic representation of main inherited cardiomyopathies presenting with atrial fibrillation. Top panels depict cartoons of hypertrophic cardiomyopathy (**A**), dilative cardiomyopathy (**B**), and arrhythmogenic cardiomyopathy (**C**); bottom panels represent exemplificative electrocardiograms of patients affected by each one of the cardiomyopathies listed above. For each disease, displayed lead is precordial unipolar V1; atrial fibrillation is the predominant cardiac rhythm in all cases, with paced QRS complexes in the tracing of a patient with severe left ventricular dysfunction, and a ventricular extra-systolic beat in the third ECG.

The common onset of AF is associated with an unfavorable prognosis (Figure 4A), with multifactorial causes, including anatomical and hemodynamic alterations related to HoCM and genetic factors. Additionally, AF derives from adverse processes of atrial remodeling, both mechanical and electrical, especially from progressive dilation of the left atrium [11,64,74,85]. It has been shown that the left atrium size ≤ 45 mm appeared to represent the threshold value associated with substantial risk of subsequent AF development, which was evident even in asymptomatic NYHA class I patients [86].

### 4.6. Dilated Cardiomyopathy

Dilated cardiomyopathy (DCM) is a non-ischemic heart muscle disease characterized by structural and functional myocardial abnormalities. This phenotype derives from dilation of the left ventricular chamber, or both, and systolic dysfunction, in the absence of coronary artery disease, hypertension, primary valvular, or congenital heart diseases [29]. Ventricular enlargement forms the basis of a progressive heart disease that culminates in HF.

Affected patients are generally at risk of arrhythmias, hospitalizations, and heart transplantation. Initial manifestations may include atrial and/or ventricular arrhythmias, eliciting death from arrhythmias, progressive HF, or both [87].

A common complication is AF, contributing to clinical deterioration, with a consequent increase in mortality and morbidity (Figure 4B). The pathophysiological processes leading to the development of AF are highly complex and may involve specific disease-related genetic mutations [Table 3] or non-specific structural changes in cardiac chambers. In individuals with DCM, ECG typically show abnormalities, with isolated changes in the T wave observed in the septal area in the presence of extensive fibrosis in the left ventricle. Delays in atrioventricular conduction and bundle branch blocks are also commonly observed. The presence of AF can lead to manifestations such as sinus tachycardia and supraventricular arrhythmias [88]. 

### 4.7. Arrhythmogenic Cardiomyopathy 

Arrhythmogenic cardiomyopathy (ACM) is a genetic disease of the myocardium, characterized by autosomal dominant inheritance, with variable penetrance and incomplete expression [89]. Although rare autosomal recessive forms, such as Carvajal syndrome and Naxos disease, have also been described, ACM is considered one of the main causes of SCD among young people and athletes. AF has been observed in 14% of patients with defined ACM and is correlated with the severity of the disease phenotype, indicating involvement of the atrial myocardium in disease progression (Figure 4C) [90].

Studies have also suggested a similar etiology between ACM and BrS. The alterations in cell–cell communication proteins can lead to changes in sodium channel structures and subsequently impact sodium handling in myocardial membranes, leading to arrhythmia [91]. ACM is known to induce cardiac remodeling resulting in atrial dilation. To this regard, alteration of the plakophilin-2 gene has been shown to affect sodium currents, further contributing to conduction abnormalities. Consequently, the risk of atrial arrhythmias increases significantly.

## 5. Ethanol-Induced AF

The term “Holiday Heart Syndrome” (HHS) is used to describe the occurrence of cardiac arrhythmias following a period of binge drinking, often observed during weekends and holidays [20,92]. The consumption of alcohol is associated with autonomic activation, recalling both sympathetic and vagal responses. Vagal activation, a characteristic of the parasympathetic system, can shorten atrial refractoriness, promoting reentry. On the other hand, sympathetic activation can increase calcium concentrations within cardiac cells. 

There is a crucial involvement of the JNK2/CaMKII pathway activation and its impact on the diastolic calcium handling by cardiac myocytes in the development of paroxysmal AF induced by excessive alcohol consumption. In rat atria exposed to ethanol and its metabolite acetaldehyde, an upregulation in the protein expression of the acetylcholine-sensitive potassium channel Kir3.1 (I KACh) has been observed. The increased I KACh activity shortens the action potential, promoting repolarization [93]. Alcohol directly affects atrial excitation–contraction coupling and may contribute to the formation of fibrotic tissue. In studies conducted on rats exposed to alcohol for two months, a decrease in myofilament sensitivity to calcium and a weaker response to inotropes were observed. Additional changes were noted at the ultrastructural level in animals that had consumed alcohol for over a year, including localized dilation and cystic alterations in intercalated discs [92,93]. Although habitual excessive alcohol consumption and binge drinking are closely associated with AF, several meta-analyses have raised the question of a relationship between habitual low to moderate alcohol consumption and a dose-dependent risk of AF, concluding that it may follow a J-shaped curve [94,95]. In a prospective multicenter observational study, consecutive patients undergoing transcatheter AF ablation were asked to limit alcohol consumption to <20 g/week after ablation. Those who reduced their consumption by at least 1% (with a median reduction of 75%) demonstrated a significantly reduced risk of AF recurrence at one year by 37% compared to patients who did not reduce the intake [96]. In addition, moderate drinking in patients with AF is linked to a noteworthy enlargement of the left atrium and a compromised transport function. On the other hand, remodeling of the atrium after transcatheter edge-to-edge repair reduces AF burden over long follow-up in patients with mitral valve disease due to multiple etiologies [97].

Overall, a positive correlation emerged between high cumulative alcohol consumption and an elevated risk of AF in apparently healthy young adults aged 20 to 39 within a national cohort in Korea [98]. Alcohol consumption, in a dose-dependent manner, not only increases the incidence of AF but simultaneously heightens the risk of thromboembolism, including ischemic stroke. 

## 6. Anticoagulation in Low Thromboembolic Risk

The CHA_2_DS_2_VASc risk score [2] confers two points for individuals aged more than 75 years, or one point when the age range is 65–74; therefore, young women (CHA_2_DS_2_VASc risk score = 1), or men (CHA_2_DS_2_VASc risk score = 0) without other risk factors are considered at minimal risk for stroke and should not be treated with oral anticoagulation (OAC) since these drugs, in this particular scenario, likely outweigh the risk of bleeding. On the other hand, AF studies addressing the risks of OAC and thrombosis account for only a small percentage of subjects with the above-mentioned features; even when a sole risk factor is added, yet benefits of OAC are unclear, thus opening a field of investigation requiring randomized clinical trials to fill the evidence gap in such heterogeneous population [99]. 

## 7. Subclinical AF in the Young

The role of wearables and the possibilities that previously implanted devices for cardiac rhythm monitoring could detect AF is highly debated, since such incidental findings might bring additional considerations on whether to recommend OAC [100].

The use of advanced technologies, such as wearable devices and artificial intelligence (AI)-based algorithms, are raising to earlier recognizing AF.

Mobile health (mHealth) technologies are becoming essential in clinical practice for the monitoring and management of cardiac arrhythmias, especially AF. These devices include smartwatches, portable ECG devices, and other lesser-known tools such as patches, belts, t-shirts, glasses, or rings. Basically, these devices work either on photoplethysmography (PPG), a technique based on light absorption and reflection of capillaries, and signals from ECG which provides single- or multiple-lead ECG data [101]. 

Furthermore, recent studies such as the Apple Heart Study and the Huawei Heart Study involved hundreds of thousands of individuals and demonstrated the effectiveness of PPG sensors integrated into smartwatches in detecting heartbeat irregularity [102,103].

Long-term ECG monitoring through devices like vests or belts have emerged as highly promising approaches for detecting AF, overcoming the limits of traditional Holter monitors. There are two main strategies: external loop recorders that capture brief ECG segments near triggering events, and mobile cardiac telemetry that transmit ECG data in real-time or intermittently to a control station. It is feasible to use adhesive patches attached to the patient’s chest instead of electrodes, although in patients with infrequent symptoms, wearable devices with limited recording time may not be sufficient to confirm a diagnosis. However, a doctor-confirmed 12-lead ECG or a 30 s single-lead ECG is mandatory to establish AF diagnosis [2].

## 8. Rhythm Control vs. Rate Control in Young Patients

Owing to long term adverse effects of antiarrhythmic drugs (AADs), chronic use of amiodarone and/or other AADs is problematic in young individuals. Following lifestyle modifications, upstream therapy with angiotensin converting enzyme inhibitors, angiotensin II type 1- and mineralocorticoid-receptors antagonists, and statins could prevent new-onset AF while ameliorating structural atrial remodeling [2]. Results with rate control drugs are somehow conflicting since calcium channel blockers do not seem to improve quality of life. Remarkably, quinidine in BrS was recommended for symptomatic AF more often than non-sustained ventricular tachycardia and history of syncope [54,104]. In addition, selective β_1_-blockade (with atenolol) on adrenoceptor cross-sensitization has been tested in human atria indicating that nonspecific β-blockade (nadolol, propranolol) is to be preferred [85]. This can be explained based on relative abundance of β-adrenoceptors and their cross regulation in the heart. On the other hand, pacing therapies seem promising. Most studies have included older patients with limited life expectancy. For younger patients, ablation of the atrioventricular node (AVN) should only be considered if there is urgent need for rate control and all other pharmacological and non-pharmacological treatment options have been carefully considered. The choice of pacing therapy (right ventricular or biventricular pacing) will depend on patient characteristics. Left bundle branch area pacing after AVN ablation may evolve as an attractive alternative pacing mode [105].

## 9. Pre-Excited AF

Ventricular pre-excitation through an accessory pathway (AP) is often asymptomatic in the young. ESC guidelines on management of Wolff-Parkinson-White syndrome consider evaluating the shortest pre-excited RR interval (SPERRI) during AF; indeed, patients with Wolff-Parkinson-White syndrome and AF are at risk of fast ventricular rates resulting from rapid conduction of atrial electrical activity to the ventricles via the AP, and at increased risk of ventricular fibrillation and sudden death. Electrical cardioversion should be readily available for hemodynamically compromised patients with pre-excited AF, and AVN modulating drugs (e.g., verapamil, beta-blockers, digoxin) should be avoided. Pharmacological cardioversion can be attempted using ibutilide, whereas class Ic AADs (procainamide, propafenone, flecainide) should be used with caution owing to their effect on the AVN [1,2,3]. Amiodarone may not be safe in pre-excited AF as it may enhance pathway conduction. Catheter ablation is recommended in asymptomatic patients in whom electrophysiology testing with the use of isoprenaline identifies high-risk properties, such as SPERRI ≤ 250 ms, AP refractoriness ≤ 250 ms, multiple APs, and an inducible AP-mediated tachycardia.

## 10. Ablative Strategies

The choice of rhythm control in AF seems the most reasonable one for preventing detrimental effects on LA remodeling and progressive reduction of cardiac function. 

Several studies demonstrated that early catheter ablation as a rhythm-control strategy in patients with paroxysmal AF may limit the progression of AF and improve clinical outcomes [1,2,3]. Since the risk of recurrent AF after ablation has been documented as well (56.5% at 36 months), currently the best therapeutic strategy seems the combination of AADs and catheter ablation. Indeed, a randomized, controlled trial has shown that in patients free from AF at the end of ablation, the continued use of previously ineffective AADs significantly reduced the recurrence of AF—a finding that AADs and ablation may have additive or being complementary [106]. On the other hand, extended interventional procedures targeting non-pulmonary vein triggers (such as ganglionated plexus block, antrum disconnection, and complex fractionated atrial electrograms isolation) represent additional effort to maintain sinus rhythm after catheter ablation in young AF patients compared to the old [107]. Finally, more emphasis on lifestyle modification, early interruption of AF, or performing non-AF substrate study could be possible therapeutic options. 

## 11. Conclusions and Future Perspectives

The occurrence of AF in the young subject requires a general multisystemic approach. While establishing the detrimental factor that triggers the AF, such as inflammation, autoimmunity, or genetic disorder, a combined therapy including OAC and rhythm control through AADs or catheter ablation needs to be considered to avoiding disease progression and preventing comorbidities. Selective atrial cardiomyopathies (FAF and SSS) fall within the expanding context of personalized medicine. Advances in genetics and the abundance of new data are driving researchers to explore the potential clinical value of AI by developing predictive models and tools for accurate AF discrimination from other tachycardias to allow tailored management strategies.

## Figures and Tables

**Table 1:** Potassium channels mutations and atrial fibrillation.

Gene	Locus	Product and Function	Known Mutations	Ref.
*KCNH2*	7q36.1	Also known as the human Ether-à-go-go-Related Gene or hERG, it encodes Kv11.1, a tetrameric ion channel conducting IKr in the heart	N588K g-o-f mutation induces abnormal QT intervals, leading to SQTS and AF L532P mutation contributes to AF by significantly shortening APD Reduced current amplitudes cause QT interval prolongation, T-wave abnormalities, and tachyarrhythmias, forming a pathophysiological substrate for LQTS2	[31]
*KCNN3*	1q21.3	It encodes KCa2.3, a calcium-activated potassium channel linking intracellular calcium to membrane potassium conductance	Rs13376333 Snp is associated with lone AF Overexpression of the SK3 channel increases the risk of SCD, bradyarrhythmias, heart block, AF and AVN dysfunction	[33,34]
*KCNJ2*	17q24.3	It encodes the inward rectifier K^+^ channel Kir2.1, which is expressed in skeletal and cardiac muscle; important contributor to the inward rectifier K^+^ current (IK1)	D172 g-o-f mutation accelerates repolarization, causing SQTS3 V93I mutation may contribute to AF by enhancing inward rectifier K^+^ channel activity D71V l-o-f mutation reduced Kir2.1 prolongs APD, induces DADs with low extracellular K^+^, dependent on Na^+^/Ca^2+^ exchanger, causing spontaneous arrhythmias and LQTS7	[35,36]
*KCNJ5*	11q24.3	It encodes Kir3.4, an inward rectifying potassium channel crucial for I KAch, regulating cardiac electrical activity in response to acetylcholine	G387R variant causes LQTS13 by disrupting KACh channel D262G variant triggers repolarization heterogeneity Two KCNJ5 SNPs (c.171C>T and c.810G>T) independently associated with early-onset lone AF in Han Chinese and Caucasian populations, linked to shortened APD and reduced RP	[37]
*KCND3*	1p13.2	It encodes Kv4.3, the alpha subunit of the voltage-gated fast transient outward potassium channel (Ito), which is found in heart and brain	A545P mutation in Kv4.3 shortens atrial APD, causing AF without heart disease in young patients BrS mutations (L450F, G600R) show T-wave changes in leads V1–V3 due to increased Ito current density	[38]
*KCNA5*	12p13.32	It encodes KV1.5, the α-subunit of the potassium channel responsible for the voltage-gated atrial-specific potassium current, IKur	E48G, A305T, D322H g-o-f mutations induce AF by reducing APD and increasing atrial tissue excitability E375X, T527M, A576V, E610K l-o-f mutations prolong APD, leading to electrical instability and AF susceptibility	[39,40,41]
*KCNE2*	21q22.11	It encodes the potassium channel beta-2 protein, Kv7.2 which interacts with HERG forming IKr, modulating ventricular repolarization; it also reduces HCN pacemaker channels and the cardiac voltage-dependent calcium channel Cav1.2 activities	I57T g-o-f variant in BrS enhances Ito current, losing epicardial AP dome R27C, M23L, and I57T l-o-f mutations are associated with early-onset familial AF Q9E, M54T, I57T variants cause a loss of IKr function and are associated with LQTS	[42]
*KCNE3*	11q13.4	It encodes the β-subunit 3 of the voltage-gated potassium channel, Kv7.3, that can interact with both Kv7.1 and Kv4.3 channels	V17M g-o-f mutation is linked to early-onset lone AF, enhancing Kv4.3/KCNE3 and Kv11.1/KCNE3 currents, increasing susceptibility to faster repolarization and atrial reentrant waves R99H mutation increases peak current density, accelerates inactivation kinetics, contributing to BrS	[43]
*KCNE4*	2q36.1	It encodes the β-subunit 4 of the voltage-gated potassium channel Kv7.3, modulates currents in channels Kv7.4 and Kv7.1 in the heart, with no effect on Kv7.5; highly expressed in the human heart	SNP rs12621643 is associated with susceptibility to AF; Inherited sequence variants in KCNE4 and KCNE5 may predispose to cardiac arrhythmias by increasing IKs activity creating a substrate for susceptibility to AF	[44]
*KCNE5*	Xq23	It encodes Kv7.5 β-subunit, shifting KCNQ1 activation by +140 mV, rendering it non-functional at physiological potentials. KCNE5 also has temperature-dependent effects on KCNQ1 gating kinetics	It suppresses the slow delayed rectifier K^+^ current (IKs), but the L65F mutation lacks this suppression, resulting in a GOF effect on IKs and predisposing to AF	[45,46]
*KCNE1*	21q22.12	It encodes the minK β subunit, combining with KCNQ1 to form the IKs potassium channel	G25V and G60D g-o-f mutations enhance IKs, shortening cardiac AP and refractory period, raising AF risk p. Asp85Asn and p. Arg36His l-o-f variants reduce by 47% IKs peak current density in the heterozygous state and is associated with LQTS5	[47]
*KCNQ1*	11p15.5-p15.4	It encodes the alpha subunit forming pores for the IKs, crucial for cardiac repolarization; it interacts with beta subunits KCNE1, KCNE2, and KCNE3	S140G, V141M, G229D, Q147R R14C, S209P, R231C and R231H g-o-f mutations cause AF F279I, V307L, V141M, R259H mutations cause SQTS2 Increased IKs shortens RP, promoting AF and ventricular arrhythmia S140G, Q147R, R231C, and R231H mutations have been associated with borderline QT prolongation KCNQ1 l-o-f mutations, such as A150T and L374H, reduce IKs, causing LQTS1	[32]
*KCNJ8*	12p12.1	It encodes Kir6.1 subunit of KATP inward rectifying potassium channel, and facilitates non-voltage-gated inward rectifying potassium current, shortening APD during metabolic stress	S422L g-o-f mutation shortens ventricular repolarization. As K-ATP channels exist in atria and ventricles, a KCNJ8 mutation can shorten atrial AP, inducing AF	[48]
*HCN4*	15q24.1	It encodes HCN4 channel subunit with slow kinetics, necessary for cardiac pacemaking process	p. Pro257Ser l-o-f variant causes early-onset AF by impairing channel function through defective membrane trafficking Polymorphisms rs498005 and rs7164883 are significantly linked to AF risk	[49]
*ABCC9*	12p12.1	It encodes SUR2, the regulatory part of KATP in cardiac, skeletal, and VSMCs; while KCNJII forms the pore, ABCC9 is essential for activation and regulation	Thr1547Ile l-o-f mutation induces KATP channelopathy, predisposing to adrenergic AF from the vein of Marshall	[50]

List of abbreviations: AF, Atrial fibrillation; APD, Action potential duration; AVN, Atrioventricular node; BrS, Brugada syndrome; DADs, Delayed afterdepolarizations; g-o-f, gain of function; l-o-f, loss of function; LQTS, Long QT syndrome; RP, Refractory period; SCD, Sudden cardiac death; SK3, Small conductance calcium-activated potassium channel 3; SNPs, Single nucleotide polymorphisms; SQTS, Short QT syndrome; SUR2, sulfonylurea receptor 2; ATP, adenosine triphosphate.

**Table 2:** Sodium channels mutations and atrial fibrillation.

Gene	Locus	Product and Function	Known Mutations	Ref.
*SCN5A*	3p22.2	It encodes Na v 1.5, the alpha subunit of the major cardiac sodium channel, which regulates the internal sodium current (INa) for rapid depolarization and initiation of excitation-contraction coupling	D1275N g-o-f variant enhances ectopic activity, increases atrial APD and excitability R1898H variant is linked to ACM reducing Na^+^ current density, potentially slowing conduction, and increasing necrosis and fibrosis E1784K variant is associated with both BrS1 and LQT3 Overlap exists between AF and other SCN5A-related diseases (LQTS3, BrS, conduction disease) H558R, R34C, S1103Y, L812Q, K817E l-o-f variants decrease sodium current density and refractory period, reducing atrial conduction velocity and increasing vulnerability to AF	[29,33,54,55]
*SCN10A*	3p22.2	It encodes Nav1.8, the voltage-dependent sodium channel responsible for late Na^+^ current in cardiomyocytes, expressed in dorsal root ganglia	Variant 1073 g-o-f increases AF risk and recurrence after catheter ablation. It enhances peak and sustained current, slowing rapid inactivation R1588Q causes a negative shift in steady-state inactivation, reducing channel availability; V94G does not conduct current; both variants in lone AF Y158D, R814H, Y158D, R814H, A1886V rare l-o-f variants may increase AF susceptibility by modulating Nav1.8 current	[56,57]
*SCN1B*	19q13.11	It encodes the β1 subunit for voltage-gated sodium channels (Nav1.5), which participates in forming the central pore; modulates channel voltage dependence, gating, cell surface expression, and cell–cell/matrix adhesion	R85H and D153N l-o-f variants reduce Na^+^ amplitude in AF R214Q increases Kv4.3 current and decreases Na^+^, heightening susceptibility to both BrS and early onset solitary AF	[58]
*SCN2B*	11q23.3	It encodes the β2 subunit for voltage-gated sodium channels (Nav1.5), covalently linked to α through disulfide bonds; regulates cardiac excitability by controlling voltage-dependent Na^+^ channel levels on the cell surface and I Na density	R28W and R28Q l-o-f variants reduce Na^+^ peak current amplitude Asp211Gly missense may be responsible for BrS decreasing cell surface expression of Nav1.5 with concomitant reduction of INa	[58,60]
*SCN3B*	11q24.1	It encodes the β3 subunit of the voltage-gated sodium channel, NaVβ3; slightly modifies Na^+^ gating	c.-324C>A enhances transcriptional activity and increases SCN3B expression, inducing AF A103V l-o-f mutation induces lone AF, while the R6K and M161T mutations are associated with early onset AF L10P mutation reduces peak current density, accelerates inactivation, slows reactivation, causing I Na loss; V110I mutation impairs Nav1.5 trafficking, reducing Na^+^ current; both involved in BrS	[62]
*SCN4B*	11q23.3	It encodes the β4 subunit of the voltage-gated sodium channel, NaVβ4; modulates channel gate kinetics, inducing negative shifts in activation voltage for certain alpha sodium channels, with no impact on inactivation voltage dependence	L179F missense mutation enhances Na^+^ channel function, causing LQTS10 p. V162G and p. I166L g-o-f mutations increase cellular excitability, forming an arrhythmogenic matrix prone to AF in 2 families Increased AF risk in p. Gly8Ser p. Leu179Phe variant generates persistent late INa in childhood LQTS p. Ser206Leu variant generates late persistent Na I and positive inactivation shift Two heterozygous variants, p. Val162Gly and p. Ile166Leu, were identified in FAF p. Thr211Arg variant was identified in a patient with AF	[63,64]

List of abbreviations: ACM, Arrhythmogenic cardiomyopathy; AF, Atrial fibrillation; APD, Action potential duration; BrS, Brugada syndrome; FAF, Familial atrial fibrillation; g-o-f, gain of function; l-o-f, loss of function; LQTS, Long QT syndrome.

**Table 3:** Cytoskeletal proteins mutations and atrial fibrillation.

Gene	Locus	Product and Function	Known Mutations	Ref.
*GJA1*	6q22.31	It encodes Connexin-43, highly expressed in atrial and ventricular myocardium	Frameshift l-o-f mutation caused by a single nucleotide deletion (c.932delC) causes AF	[74]
*GJA5*	1q21.2	It encodes Connexin-40 which cooperates with Cx-43 in mediating propagation of impulses	Q236H l-o-f mutation is linked to reduced gap junction activity and AF p. A96S mutation is associated with lower junctional conductance and increased voltage sensitivity	[72,73]
*NUP155*	5p13.2	It encodes Nucleoporin 155, essential part of the nuclear pore complex involved in protein binding and translocation during nucleocytoplasmic transport; embryogenesis	p. R391H l-o-f mutation affects the nuclear localization of NUP155 and reduces the permeability of the nuclear envelope leading to a reduced AP	[70]
*MYL4*	17q21.32	It encodes an alkaline myosin light chain, specifically expressed in the human atria post-birth	Rare frameshift l-o-f deletion in the myosin gene MYL4 (c.234delC) found in early onset AF	[80]
*MYH6*	14q11.2	It encodes the cardiac myosin alpha heavy chain subunit (alpha-MHC), a fast ATPase expressed primarily in atrial tissue	p. R721W missense variant found in AF and sick sinus syndrome three SNPs (rs28730771, rs365990, and rs2277473) are significantly associated with the risk of susceptibility to AF	[75]
*LMNA*	1q22	It encodes lamins A and C, key components of the nuclear lamina, playing crucial roles in nucleus structure, chromatin organization, gene regulation, and DNA repair	p. Arg377Leu and p. Arg377Cys missense in AF	[76]
*SYNE2*	14q23.2	It encodes Nesprin-2, linking organelles to actin for subcellular organization, and part of LINC for interaction nuclear lamina-cytoskeleton	+ 688G mutation is associated with AF rs1152591 and rs1152595 SNPs downregulate SYNE2α1 expression, increasing nuclear-cytoskeletal connectivity and nuclear rigidity in AF	[75]
*DES*	2q35	It encodes Desmin, a classical type III intermediate filament protein, which maintains structural/mechanical integrity of atrial cardiomyocytes sinoatrial node, atrioventricular node, and His-Purkinje system	Altered Desmin leads to cytoskeletal defects, aggregation, and abnormal Ca^2+^ distribution and arrhythmias	[77]
*JPH2*	20q13.12	It encodes Junctophilin 2, membrane-binding protein that binds plasma membrane and SR for excitation-contraction coupling	E169K mutation can lead to AF through aberrant release of Ca^2+^ from the SR mediated by loss of binding with RyR2	[78]
*TTN*	2q31.2	It encodes Titin, a molecular scaffold for sarcomere assembly and signaling involved in DCM	Truncating rare variants are linked to FAF	[79]
*MYH7*	14q11.2	It encodes beta-cardiac/skeletal slow myosin heavy chain (MyHC-slow), expressed predominantly in cardiac ventricles and slow skeletal myofibers (type 1)	MYH7 gene variants are associated with HCM and DCM and increase the likelihood of AF	[75]

List of abbreviations: AF, Atrial fibrillation; AP, Action potential; DCM, Dilated cardiomyopathy; FAF, Familial atrial fibrillation; HCM, Hypertrophic cardiomyopathy; l-o-f, loss of function; RyR2, Ryanodine receptor 2; SR, Sarcoplasmic reticulum.

## Data Availability

Source of data is the project of the Italian Ministry of Health: Inherited arrhythmias: clinical characterization, genetic geography and experimental studies in the Calabria Region isolate—RF-2011-02348444.
